# Comparison of the responsiveness of the Foot Health Status Questionnaire and the Manchester Foot Pain and Disability Index in older people

**DOI:** 10.1186/s12955-014-0158-4

**Published:** 2014-10-25

**Authors:** Hylton B Menz, Maria Auhl, Sonja Ristevski, Nicoletta Frescos, Shannon E Munteanu

**Affiliations:** Lower Extremity and Gait Studies Program, School of Allied Health, La Trobe University, Bundoora, 3086 Victoria Australia; Department of Podiatry, School of Allied Health, La Trobe University, Bundoora, 3086 Victoria Australia

**Keywords:** Foot, Health status, Questionnaires, Responsiveness

## Abstract

**Background:**

In recent years, several questionnaires have been developed for the assessment of foot health and its impact on quality of life. In order for these tools to be useful outcome measures in clinical trials, their ability to detect change over time (responsiveness) needs to be determined. Therefore, the aim of this study was to assess the responsiveness of two commonly-used questionnaires in older people with foot pain.

**Methods:**

Participants (n = 59; 24 women and 35 men, mean age [SD] 82.3 [7.8] years) allocated to the intervention arm of a randomised controlled trial assessing the effectiveness of extra-depth footwear compared to usual care completed the Foot Health Status Questionnaire (FHSQ) and Manchester Foot Pain and Disability Index (MFPDI) at baseline and 16 weeks. Responsiveness of the FHSQ subscales (pain, function, footwear and general foot health) and MFPDI subscales (pain, functional limitation and concern about appearance) was determined using (i) paired *t*-tests, (ii) Cohen’s *d*, (iii) the standardised response mean (SRM), and (iv) the Guyatt index.

**Results:**

Overall, the FHSQ pain subscale exhibited the highest responsiveness, as evidenced by a highly significant paired *t*-test (*p* <0.001), Cohen’s *d* = 0.63 (medium effect size), SRM = 0.50 (medium effect size) and Guyatt index = 1.70 (huge effect size). The next most responsive measure was the FHSQ function subscale, as evidenced by a borderline paired *t*-test (*p* = 0.050), Cohen’s *d* = 0.37 (small effect size), SRM = 0.26 (small effect size) and GI = 1.22 (very large effect size). The FHSQ footwear, FHSQ general foot health and MFPDI pain, functional limitation and concern about appearance subscales demonstrated lower responsiveness, with negligible to medium effect sizes.

**Conclusion:**

The FHSQ pain and function subscales were most responsive to change in older people with foot pain receiving a footwear intervention. These findings provide useful information to guide researchers in selecting appropriate outcome measures for use in future clinical trials of foot disorders.

## Background

Foot pain affects one in four people over the age of 45 years [1] and has a significant impact on mobility [2,3] and quality of life [4,5]. In recent years, several patient-reported outcome measures have been developed to assess the severity and impact of foot pain in clinical practice, epidemiological studies and clinical trials [6-11]. In order to provide useful information, these questionnaires need to be both valid (i.e. they actually measure what they are intended to measure) and reproducible (i.e. they are able to produce the same scores in identical conditions on different occasions). In addition, to be useful outcome measures in clinical trials, such tools need to be capable of detecting changes in health status over time, a construct commonly referred to as ‘responsiveness’ [12-16].

Broadly speaking, there are two main approaches for assessing the responsiveness of an outcome measure, most commonly referred to as *anchor-based* and *distribution-based* [17]. *Anchor-based* approaches compare interval changes in outcome measure scores to a dichotomised ‘global’ rating of change score (using a question such as “Overall, how has your condition changed as a result of your treatment?”). The outcome measure score that corresponds to a meaningful change (generally defined as a response of “somewhat better” or above) is considered to be the smallest difference which participants perceive as beneficial, and is termed the minimal important difference, or MID [12]. The MID can then be used as a benchmark for interpreting the effectiveness of an intervention. *Distribution-based* approaches involve examining the statistical distribution of change scores for interval outcome measures (for either between-group or within-group change over time comparisons), and applying a range of statistics to calculate effect sizes [18]. The smaller the MID or larger the effect size, the more responsive an outcome measure is considered to be.

Recent reviews of measures of foot function, foot health and foot pain indicate that few have undergone adequate evaluation of responsiveness, thereby limiting their use in clinical trials [6-11]. Two of the most commonly used and most extensively validated measures of foot pain and disability are the Foot Health Status Questionnaire (FHSQ) [19] and the Manchester Foot Pain and Disability Index (MFPDI) [20], but neither has undergone detailed analysis of responsiveness. In a study of people with plantar heel pain receiving foot orthoses, the FHSQ was shown to be more responsive than the Foot Function Index (FFI), based on the observation of significant improvements in all four subscales of the FHSQ but only two out of three subscales of the FFI [21]. No responsiveness data have been published for the English language version of the MFPDI, although a modification of the MFPDI – the Manchester-Oxford Foot Questionnaire – has been shown to be responsive to improvements in foot health status following hallux valgus surgery [22], and a recent study concluded that a Dutch version of the MFPDI demonstrated only moderate responsiveness [23].

Given the increasing use of the FHSQ and MFPDI in foot and ankle research, there is a need for a more detailed evaluation of responsiveness of these instruments in order to determine whether it is appropriate to employ them as outcome measures in clinical trials of interventions for foot disorders. Therefore, as part of a randomised controlled trial assessing the effectiveness of extra-depth footwear in older people with foot pain [24,25], we compared the responsiveness of the FHSQ and MFPDI subscales, using a range of recommended statistical approaches [12-16].

## Methods

### Study design

This study was undertaken as part of a larger randomised controlled trial, the details of which have been published previously [24,25]. Briefly, the trial was a parallel-group randomised controlled trial design with a 16-week follow-up period, with participants randomly allocated to either a ‘usual care’ control group or the intervention group. The intervention group was provided with off-the-shelf footwear at the baseline assessment, and the data obtained from this group form the basis of the current study.

### Participants

Participants residing in Melbourne, Victoria, Australia were recruited from the Department of Veterans’ Affairs (DVA) database between October 2012 and May 2013. To be eligible to be included in the study, veterans needed to: (i) be aged 65 years or over; (ii) be a current DVA Gold Card client not eligible for medical grade footwear; (iii) have received podiatry treatment on at least three occasions in the past five years; (iv) have disabling foot pain, using the case definition of the MFPDI [20] proposed by Roddy *et al*. [26]; (v) have persistent foot pain, defined as foot pain present for at least 12 weeks, and; (vi) be capable of understanding the English language in verbal and written form. Participants were deemed ineligible for inclusion if they: (i) were currently residing in a high level care residential aged care facility; (ii) had diabetes and a history of foot ulceration (or current foot ulceration) or diabetic peripheral neuropathy (diagnosed with the 5.07 Semmes-Weinstein monofilament, using the International Working Group on the Diabetic Foot protocol [27]); (iii) had a neurodegenerative disorder (e.g. Parkinson’s disease); (iv) had a lower limb or partial foot amputation (although single toe amputations will be permitted); (v) had been prescribed contoured foot orthoses within the past 3 months; (vi) were currently wearing the intervention footwear, or; (vii) had cognitive impairment (defined as a score of <7 on the Short Portable Mental Status Questionnaire [28]).

The Australian DVA Human Research Ethics Committee provided ethical approval (approval number E012/005[5.1]) and the La Trobe University Human Ethics Committee formally accepted this approval (E012/004). All participants provided written informed consent prior to enrolment.

### Intervention

The intervention group was provided with off-the-shelf, extra-depth footwear (Dr Comfort®, Vasyli Medical, Queensland, Australia). Men received the *Brian* style and women received the *Annie* style. Both styles were available in three width fittings (medium, wide, extra-wide) and featured a stretchable Lycra® upper with Velcro® closure and a choice of two removable insoles (a flat, 4 mm foam insole or a cushioning insole with a contoured heel cup, 7 mm thick under the forefoot and 15 mm thick under the heel). The shoes were lightweight (ranging from approximately 200 to 500 gm, depending on size) and meet all commonly-used criteria for appropriate footwear for older people with foot problems [29-33]. The assessors measured participants’ feet using a Brannock Device® (Brannock Device Co, Inc., Liverpool, New York, USA) to ensure appropriate length and width, using the fitting protocol recommended by the footwear manufacturer [34]. Intervention group participants who wore flat insoles (or had been wearing contoured foot orthoses for more than 3 months) in their current footwear were permitted to wear them in their study footwear, provided that the fit of the shoes was appropriate. The accuracy of the shoe fitting procedure was evaluated by comparing total length, forefoot width and forefoot girth measurements of the foot obtained with a three-dimensional scanner to the corresponding last measurements of the allocated shoe size provided by the manufacturer, and participants reported the shoes to be well fitted and comfortable [35].

### Foot Health Status Questionnaire (FHSQ)

The FHSQ consists of 13 questions reflecting four foot health-related domains: pain (4 questions), function (4 questions), footwear (3 questions), and general foot health (2 questions) [19]. Each question (item) is scored on a 5-point Likert scale, and individual item scores are then re-coded, tabulated, and finally transformed to a scale ranging from 0 (indicating poorest foot health) to 100 (indicating best foot health) for each of the 4 domains. The FHSQ demonstrates a high degree of content, criterion, and construct validity and high retest reliability, and has been used as an outcome measure in clinical trials for a range of foot disorders [9]. The FHSQ was measured at baseline and at 4, 8, 12 and 16 weeks. For the purpose of the analysis in this study, only the baseline and 16 week scores were used.

### Manchester Foot Pain and Disability Index (MFPDI)

The MFPDI (Isis Innovation Ltd., Oxford, UK) consists of 19 statements prefaced by the phrase ‘Because of pain in my feet’, formalised under three subscales: functional limitation (10 items), pain intensity (five items) and personal appearance (two items). The remaining two items are concerned with difficulties in performing work or leisure activities, which are omitted if the respondent is of retirement age. Each of the 17 statements has a three-category response: ‘none of the time’ (score = 0), ‘on some days’ (score = 1) and ‘on most/every day(s)’ (score = 2) [20]. The MFPDI has primarily been used in epidemiological studies, but has also been employed as an outcome measure in clinical trials [36,37]. The MFPDI was measured at baseline and at 4, 8, 12 and 16 weeks. For the purpose of the analysis in this study, only the baseline and 16 week scores were used. To express the MFPDI as a linear, interval scale, Rasch-transformed scores calculated from the original MFPDI dataset by Gijon-Nogueron *et al.* [38] were used.

### Statistical analysis

All data were normally distributed based on observations of linear normal Q-Q plots and skewness values of between −1 to +1. To evaluate the responsiveness of the FHSQ and MFPDI subscales, four different effect size statistics were applied [12-16]:

#### Paired t-test

The paired *t*-test was used to test the null hypothesis that there was no change in the mean scores from baseline to the 16 week follow-up. This was calculated using the formula:

$$ {t}_0=\frac{{\overline{D}}_x}{SD\left({D}_x\right)/\sqrt{n}} $$

…where ${\overline{D}}_x$ = mean change scores between baseline and follow-up*, SD(D*_*x*_*)* = standard deviation in change scores from baseline to 16 week follow-up, and *n* = sample size.

#### Cohen’s d

Also referred to as the standardised effect size, Cohen’s *d* was calculated as the mean change scores between baseline and 16 week follow-up, divided by the standard deviation of the baseline scores [39]:

$$ d=\frac{{\overline{D}}_x}{SD(X)} $$

#### Standardised response mean

The standardised response mean (SRM), also known as the responsiveness treatment coefficient [40], was calculated as the mean change scores between baseline and 16 week follow-up divided by the standard deviation of the differences between the baseline and 16 week follow-up scores:

$$ SRM=\frac{{\overline{D}}_x}{SD\left({D}_x\right)} $$

#### Guyatt index

The Guyatt index (GI) [12] represents the magnitude and variability in change scores for an outcome measure relative to the minimal important difference of the measure, and was calculated as:

$$ GI = {\Delta}_x/\sqrt{2*\ MS{E}_x} $$

…where *Δ*_*x*_ = the minimal important difference of the measure and *MSE*_*x*_ = the standard deviation of the individual change scores. In order to determine the minimal important difference for each outcome measure subscale, participants’ perceptions of the overall treatment effect were documented at the 16 week follow-up using the question “Overall, how has your foot pain changed since the start of the study?”, with a 5-point Likert scale response (“marked worsening”, “moderate worsening”, “same”, “moderate improvement” or “marked improvement”). This scale was dichotomised, with a positive outcome defined as moderate or marked improvement [24]. The minimal important difference for each measure was then calculated as the mean change score in participants who improved minus the mean change score in participants who did not improve or whose symptoms worsened.

To aid interpretation of the effect size statistics, the following benchmarks were used: negligible effect size (<0.15), small effect size (≥0.15 and <0.40), medium effect size (≥0.40 and <0.75), large effect size (≥0.76 and <1.10), very large effect size (≥1.10 and <1.45) and huge effect size (>1.45) [41].

## Results

### Participant characteristics

Postal invitations were sent to 2,457 DVA clients and 341 were screened for eligibility by telephone. Of these, 121 underwent baseline screening and were randomised: 61 into the control group and 60 into the intervention group. Of the 60 participants allocated to the intervention group, one participant withdrew consent, leaving 59 participants with complete data. Characteristics of the sample are provided in Table 1.

**Table 1:** **Participant characteristics (n = 59)**

Age, mean (SD) years	82.3 (7.8)
Sex, n (%) female	24 (40.7)
Body mass index, mean (SD) kg/m^2^	29.2 (5.4)
Major medical conditions, n (%)	
Diabetes	8 (13.6)
Heart disease	19 (32.2)
Osteoarthritis	48 (81.4)
Rheumatoid arthritis	3 (5.1)
Stroke	8 (13.6)
Use of >4 medications	50 (84.7)
Foot problems	
Foot pain duration, mean (SD) years	13.1 (14.6)
Hallux valgus, n (%)	20 (33.9)
Lesser toe deformity, n (%)	52 (88.1)
Keratotic lesions on toes, n (%)	13 (22.0)
Plantar keratotic lesions, n (%)	37 (62.7)

### Responsiveness

Means and standard deviations for the FHSQ and MFPDI subscales are shown in Table 2. The four responsiveness statistics for the FHSQ and MFPDI subscales are shown in Table 3. Overall, the FHSQ pain subscale exhibited the highest responsiveness, as evidenced by a highly significant paired *t*-test (*p* <0.001), Cohen’s *d* = 0.63 (medium effect size), SRM = 0.50 (medium effect size) and GI = 1.70 (huge effect size). The next most responsive measure was the FHSQ function subscale, as evidenced by a borderline paired *t*-test (*p* = 0.050), Cohen’s *d* = 0.37 (small effect size), SRM = 0.26 (small effect size) and Guyatt index = 1.22 (very large effect size). The FHSQ footwear, FHSQ general foot health and MFPDI pain, functional limitation and concern about appearance subscales demonstrated lower responsiveness, with negligible to medium effect sizes.

**Table 2:** **Mean (SD) scores for each outcome measure subscale at baseline and 16 week follow-up**

	**Baseline mean (SD)**	**16 week follow-up mean (SD)**
FHSQ		
Pain	54.8 (20.1)	67.4 (23.1)
Function	54.1 (22.8)	62.6 (27.9)
Footwear	30.5 (25.4)	28.2 (23.5)
General foot health	34.4 (24.4)	41.4 (26.4)
MFPDI		
Pain	5.0 (1.6)	4.6 (2.1)
Functional limitation	10.5 (2.3)	9.4 (3.3)
Concern about appearance	1.1 (1.2)	1.0 (1.3)

FHSQ: Foot Health Status Questionnaire, MFPDI: Manchester Foot Pain and Disability Index.

**Table 3:** **Responsiveness of the outcome measure subscales**

	**Paired** ***t*** **-test**		**Cohen’s** ***d***		**SRM**		**GI**	
	***t***	***p***	**Value**	**Interpretation**	**Value**	**Interpretation**	**Value**	**Interpretation**
FHSQ								
Pain	−3.83	<0.001	0.63	Medium	0.50	Medium	1.70	Huge
Function	−2.01	0.050	0.37	Small	0.26	Small	1.22	Very large
Footwear	0.89	0.378	0.09	Negligible	0.12	Negligible	0.21	Small
General foot health	−2.06	0.044	0.29	Small	0.27	Small	0.68	Medium
MFPDI								
Pain	1.61	0.114	0.26	Small	0.21	Small	0.51	Medium
Functional limitation	2.62	0.011	0.46	Medium	0.34	Small	0.41	Medium
Concern about appearance	0.61	0.542	0.09	Negligible	0.08	Negligible	0.06	Negligible

FHSQ: Foot Health Status Questionnaire, MFPDI: Manchester Foot Pain and Disability Index, SRM: standardised response mean, GI: Guyatt index.

## Discussion

The aim of this study was to evaluate the responsiveness of two commonly used measures of foot pain and disability: the Foot Health Status Questionnaire (FHSQ) [19] and the Manchester Foot Pain and Disability Index (MFPDI) [20]. To do this, we applied four of the most widely used responsiveness statistics to FHSQ and MFPDI subscale data obtained at baseline and at 16 weeks of follow-up from a clinical trial of off-the-shelf footwear for reducing foot pain in older people [24,25]. Overall, the FHSQ pain subscale exhibited the highest responsiveness, as evidenced by a highly significant paired *t*-test and effect sizes ranging from medium to huge. The next most responsive measure was the FHSQ function subscale (borderline paired *t*-test and effect sizes ranging from small to very large). Based on these findings, it would appear that the FHSQ is preferable to the MFPDI as an outcome measure in clinical trials evaluating the effectiveness of interventions in reducing foot pain and improving foot function in older people.

The FHSQ footwear and MFPDI concern about appearance subscales performed poorly in this analysis, with negligible to small effect sizes. However, these subscales are not particularly useful in the context of a trial involving a standardised footwear intervention, as the FHSQ footwear subscale items reflect difficulty with obtaining suitable footwear, and the MFPDI concern about appearance subscale items focus on participants’ self-consciousness regarding the appearance of their feet and shoes. These two subscales were included in the analysis for the sake of completeness, as the FHSQ and MFPDI are generally administered in their entirety rather than as selected subscales. The poor responsiveness reported here is therefore not a useful indicator of the potential value of these subscales when applied to other interventions. For example, Bennett *et al*. [42] have demonstrated significant improvements in the FHSQ footwear subscale following foot surgery, which is likely due to the surgery allowing a wider range of shoes to be worn postoperatively.

Our observation of the limited responsiveness of the MFPDI is consistent with van der Zwaard *et al.* [23], who reported that a Dutch translation of the MFPDI was only moderately responsive to change in people aged 50 years or over who were enrolled in a randomised trial of treatment for forefoot pain. The authors suggested several reasons for this, including: (i) the three level response options (‘none of the time’, on some days’ and ‘on most/every day/s’) are too widely spaced, (ii) pain intensity is not directly addressed, and (iii) the concern about appearance subscale (consisting of only two items) has a large floor effect. In developing the Manchester-Oxford Foot Questionnaire (a modification of the MFPDI for use in foot surgery), Dawson *et al*. [43] addressed many of these issues by increasing the response categories from three to five, adding a pain severity item, and combining the concern about appearance and ability to undertake social, recreational and work activities items into a separate construct referred to as ‘social interaction’. The high responsiveness of this amended scale in patients undergoing hallux valgus surgery [22] suggests that there may be some scope for improving the MFPDI as an outcome measure.

When interpreting these findings, it should be noted that there is currently no accepted gold standard approach for assessing responsiveness of outcome measures, and that each statistical approach has limitations (for a detailed discussion, see Husted *et al*. [13] and Revicki *et al.* [16]). Paired *t*-tests provide an indication of the statistical significance of the observed change in the outcome measure scores, but this is influenced not only by the magnitude of change, but also the sample size (i.e. larger sample sizes are more likely to detect statistically significant differences). Cohen’s *d* and the standardised response mean are influenced by the variability of the denominator (baseline scores for Cohen’s *d* and change scores for standardised response mean), so higher variability in the denominator will result in smaller effect sizes. Finally, although the Guyatt index is considered by some to be the most appropriate effect size statistic [13,44], it requires the calculation of a minimal important difference, which may vary across different populations, conditions and interventions [16]. For this reason, we determined the minimal important difference of the FHSQ subscales by dichotomising a 5-point Likert scale response of perceived overall improvement (i.e. a positive outcome defined as moderate or marked improvement) and calculating mean change scores from our data, rather than using minimal important difference scores calculated from people with heel pain reported by Landorf *et al.* [45]. As no minimally important difference scores have been reported for the MFPDI, we used the same approach for each of the MFPDI subscales, which also allowed us to make direct comparisons between the two outcome measures. However, there is currently no consensus regarding the most appropriate question or number of response levels in determining the anchor used to define the minimal important difference, and anchor-based approaches have limited discriminative ability in trials where most participants report improvement in their condition [16].

Despite these limitations, and the fact that each test calculates the magnitude of the effect size in different ways, the four statistics we used resulted in a reasonably consistent pattern of responsiveness across the outcome measure subscales. We can therefore be more confident of the superiority of the FHSQ pain and function subscales using this combined approach rather than using one statistical test alone. Nevertheless, this approach only addresses ‘internal’ responsiveness (the ability of a measure to change over time), not ‘external’ responsiveness, which Husted *et al*. [13] have defined as the extent to which changes in a measure over time relate to a corresponding change in an established reference measure of health status. We were unable to evaluate external responsiveness in this study due to the absence of an appropriate reference measure for comparison. Although we collected Short Form 12 data from this sample, generic health outcome measures are generally less responsive than condition-specific measures and are therefore not considered to be suitable reference standards [6,22]. Outcome measures more directly related to mobility and physical function in older people, such as the disability index of the Health Assessment Questionnaire [46], may be more appropriate reference standards for future evaluation of external responsiveness.

Our findings provide further support for the continued use of the FHSQ as an outcome measure in clinical trials of foot disorders. However, although the recoding of FHSQ response from a Likert scale to a 100-point scale enables the FHSQ subscales to be expressed as interval data, the FHSQ has so far not undergone Rasch analysis – a statistical technique which evaluates whether overall scores summed from ordinal items can be considered to be linear, interval-level variables [47]. Such an analysis is necessary to confirm whether it is indeed appropriate to analyse FHSQ subscales using parametric statistical approaches.

In summary, this study has shown that the FHSQ pain and function subscales are most responsive to change over time in older people receiving a footwear intervention to alleviate foot pain. The FHSQ footwear and general foot health subscales and the three subscales of the MFPDI exhibited lower responsiveness, so may not be appropriate outcome measures in this population. Further research is required to determine the internal responsiveness of the FHSQ as an outcome measure in trials of other foot disorders, interventions and clinical populations, and to evaluate external responsiveness against an appropriate reference standard.
